# A Practical Introduction to Mechanistic Modeling of Disease Transmission in Veterinary Science

**DOI:** 10.3389/fvets.2020.546651

**Published:** 2021-01-26

**Authors:** Carsten Kirkeby, Victoria J. Brookes, Michael P. Ward, Salome Dürr, Tariq Halasa

**Affiliations:** ^1^Department of Veterinary and Animal Sciences, Faculty of Health and Medical Sciences, University of Copenhagen, Frederiksberg, Denmark; ^2^School of Animal and Veterinary Sciences, Faculty of Science, Charles Sturt University, Wagga, NSW, Australia; ^3^Graham Centre for Agricultural Innovation (Charles Sturt University and NSW Department of Primary Industries), Wagga, NSW, Australia; ^4^Faculty of Veterinary Science, Sydney School of Veterinary Science, University of Sydney, Sydney, NSW, Australia; ^5^Department of Clinical Research and Public Health, Veterinary Public Health Institute, University of Bern, Bern, Switzerland

**Keywords:** simulation model, transmission model, disease dynamics, mechanistic model, disease model

## Abstract

Computer-based disease spread models are frequently used in veterinary science to simulate disease spread. They are used to predict the impacts of the disease, plan and assess surveillance, or control strategies, and provide insights about disease causation by comparing model outputs with real life data. There are many types of disease spread models, and here we present and describe the implementation of a particular type: individual-based models. Our aim is to provide a practical introduction to building individual-based disease spread models. We also introduce code examples with the goal to make these techniques more accessible to those who are new to the field. We describe the important steps in building such models before, during and after the programming stage, including model verification (to ensure that the model does what was intended), validation (to investigate whether the model results reflect the modeled system), and convergence analysis (to ensure models of endemic diseases are stable before outputs are collected). We also describe how sensitivity analysis can be used to assess the potential impact of uncertainty about model parameters. Finally, we provide an overview of some interesting recent developments in the field of disease spread models.

## Introduction

A disease spread model is a simplified representation of a real-life system of disease transmission. As defined by Lessler and Cummings (1), disease spread models (also known as mechanistic models of disease spread) include explicit hypotheses of the biological mechanisms that drive infection dynamics. Therefore, they differ from statistical models such as regression models. Disease spread models are motivated by a need to better understand the transmission dynamics of a disease, predict the spread of the disease in a population and its effects, and study how the spread can be influenced (including the evaluation of different strategies to improve surveillance and control of diseases). The quote, “all models are wrong, but some are useful,” (2) is often stated because disease spread models are simpler than reality, but they generate information which is otherwise difficult to obtain (3). For example, experiments on disease transmission and control might insufficiently represent real-life disease ecology, or not be feasible due to high resource requirements (such as time and monetary costs), or logistical and ethical constraints. In addition, observational studies of disease spread might not provide comparisons of the relevant control strategies, or not occur in the population of interest (e.g., transboundary diseases).

Models of disease transmission can represent diverse diseases, including bacterial and viral infections, as well as parasites and vector-borne diseases, in a range of host populations and environments, and at different scales (4). Disease spread models might identify critical elements and knowledge gaps by reconstructing a system using available knowledge (5). They can also be useful decision-making tools by simulating surveillance or control of a specific disease and comparing strategies in specific contexts, such as outbreak situations (6, 7). Models have also been used to inform outbreak preparedness [e.g., (8, 9)], and the control of endemic pathogens [e.g., (10–13)].

Here, we focus on modeling the spread of infectious diseases of animals in a range of contexts. The methods described are not unique to veterinary systems and are used in other disciplines such as ecology and human health. In particular, we focus on a class of model called individual-based models (IBMs). Mancy et al. (4) provide an in-depth discussion of the different motivations for developing disease spread models in ecology and animal health. They present a conceptual framework to guide model construction, focusing on the pre-modeling stage (model selection, establishing, and testing the theory). In building on Mancy et al. (4) our objectives are 3-fold; (1) to provide a practical introductory guide to the process of developing a mechanistic model of animal disease transmission using IBMs, aimed at researchers beginning in this field; (2) to describe important concepts before, during and after the programming stage of developing model of animal disease transmission; and (3) to provide practical examples of models, including code, in veterinary science. Thus, we provide a hands-on introduction to model building, and its use and challenges, for scientists starting to work on disease spread models.

## Methods

### Definitions and Concepts

Before we describe the steps of model building in the context of IBMs, we briefly describe some key terms, concepts, and approaches applied in disease spread modeling. Terminology in this field can be inconsistent; for a list of terms and definitions used throughout this guide, see Appendix 1.

#### Terms Used in Disease Spread Modeling

Disease spread models simulate the transmission of an infectious disease between the disease hosts, who are modeled as *units of interest*. This unit is the smallest entity of the model and could be an individual animal (or part of it; for example, a quarter of the udder in a mastitis model), a group of animals, herds, or populations in regions or countries. The units of interest can be aggregated and modeled as proportions of the total population in each disease state (see below) at a given time, or modeled as individuals whose disease status is tracked through the disease states included in the model.

The simulated system includes time, making the model *dynamic*. Time can be modeled as a *continuous* or *discrete* process. In the latter a fixed time-interval is chosen and the model steps through each consecutive interval (time-step) and updates the numbers of units of interest in each disease state from the beginning to the end of the simulated period (for example, every day, for a year) or until the disease fades out. In contrast, if time is modeled as a continuous process, the rate of change in the relative numbers of units of interest in each disease state in the system is continuously modeled using differential equations.

For discrete time models, the length of a time step is designated by the modeler and depends on the disease dynamics, purpose of the model (for example, predictions in monthly time-steps might be useful for surveillance or disease control), the availability of data needed to parameterize the model (outbreak data might only be available on a yearly scale), and the time spent by an individual unit of interest in each disease state of the model (see below). Whilst daily time-steps are typical for most discrete disease-spread models (11), weekly (14) or biweekly [e.g., (15)], biannual (16), or even yearly time steps can be used [for example, when simulating long duration control programs, such as (13)].

A model can be *deterministic* or *stochastic*. A model is *stochastic* when there is variation in model outputs arising from the use of distributions to describe input parameters (rather than fixed values), or by allowing model events to occur as random processes (inherent stochasticity). See section “Modeling Disease Transmission” for illustration of the difference between deterministic and stochastic. The outputs from a stochastic model will vary every time the model is run. In contrast, outputs from deterministic models are consistent each time the model is run.

**Figure 1:**
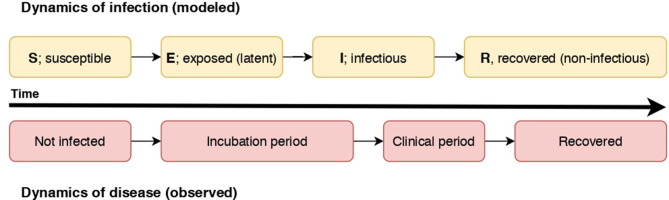
A diagram illustrating the relationship of the dynamics of an SEIR infectious process and observed disease states in individuals. In this case, individuals become non-infectious prior to resolution of clinical signs.

Disease spread models represent the dynamics of infection, or progression of the modeled units of interest through *disease states*, for instance *Susceptible (S), Infectious* (*I*), and *Recovered (R)* states (an SIR model). In a susceptible state, a unit of interest has yet to be exposed to an infectious individual and infected (termed “effective contact”). Once effective contact has occurred, an individual is in an infectious state prior to transition to a recovered state (or death). This basic formulation can be expanded with other disease states; for example, an *Exposed (E)* state representing the latent period of the infection can occur prior to transitioning to the *Infectious (I)* state [for example, within-herd spread of FMD; (17)]. The modeled states are dependent on the natural history of the disease, the purpose and scale of the model, and the resolution of available data. For example, differentiation of clinical and subclinical infectious states can be included if the subclinical state is considered significant to spread given the scale of the model, or if clinical detection of the disease is an essential aspect in the model. In a model of rabies spread, the pre-infectious period of rabies was considered essential to include in a model in which the dog populations were small (18), and not considered necessary in a similar but larger-scale model of rabies spread in dog populations in Chad (19). We illustrate how the dynamics of infection as modeled in an SEIR model relate to the dynamics of disease (the observed states) in Figure 1.

The way in which the units of interest contact each other, or how they “mix,” is a core component of a disease model. *Homogeneous* contact means that all the units have equal probability of contact with each other (no clustering). *Heterogeneous* contact means that the probability of contact between units of interest is not equal, hence clustering (spatial or related to other contact characteristics) exists in the population. Heterogeneous contact can be modeled by stratifying models into population groups (for example, by age or farm type), modeling contacts between units of interest according to a network structure, or modeling specific characteristics of units that influence contact [for example, furious rabies in dogs; (18)].

#### Modeling Approaches

Since Kermack and McKendrick first formulated the basic compartmental equation-based SIR model using differential equations in 1927 (20), numerous approaches to modeling disease transmission have been developed. For a comprehensive description of modeling approaches, see Mancy et al. (4). Briefly, models can be classified according to how the disease hosts are modeled (as individual units of interest, or as groups in which the proportion of units of interest in disease states are followed) and how contact occurs (the connectivity between units), then further differentiated on how time is modeled (discrete or continuous) and whether stochasticity is included.

Here, we focus on individual-based models [IBMs, or Individual-level models; Mahsin et al. (21)] in which individual units of interest are described and followed through the disease states. The units of interest in IBMs represent discrete entities (such as individuals or herds) and time steps are discrete.

An advantage of IBMs is that units of interest can be assigned their own properties that can influence disease transmission, detection or control. They are therefore useful to simulate heterogeneity in disease transmission between the units of interest. For example, in a model of foot-and-mouth disease (FMD), an individual herd might be predominantly either sheep or cattle, which might influence disease susceptibility and transmission at the herd level (22, 23). Agent-based models (ABMs) are a subset of IBMs in which contact—and hence disease transmission—is simulated between explicit pairs of individual units of interest. ABMs often include explicit movement of—and therefore, contacts between—individual units of interest, thus introducing contact heterogeneity in the population due to spatial variation (24). In an example in which rabies transmission was modeled, individual dogs were assigned specific roaming characteristics that influenced their contacts with other dogs (25). In a further example, heterogeneity of contacts between individuals was assigned using individuals' social network parameters (18, 19). Consequently, these models can have a high level of complexity, but also be computationally intensive (and consequently, relatively slow to implement and simulate).

If the unit of interest in an IBM is a group of individual animals (for example, herds), within-group disease spread can be modeled using an equation-based model with proportions of the unit of interest in disease-state compartments. In this case, specific individuals are not tracked. Such models are called nested models in ecological modeling (26).

### Building an Individual-Based Model

**Figure 2:**
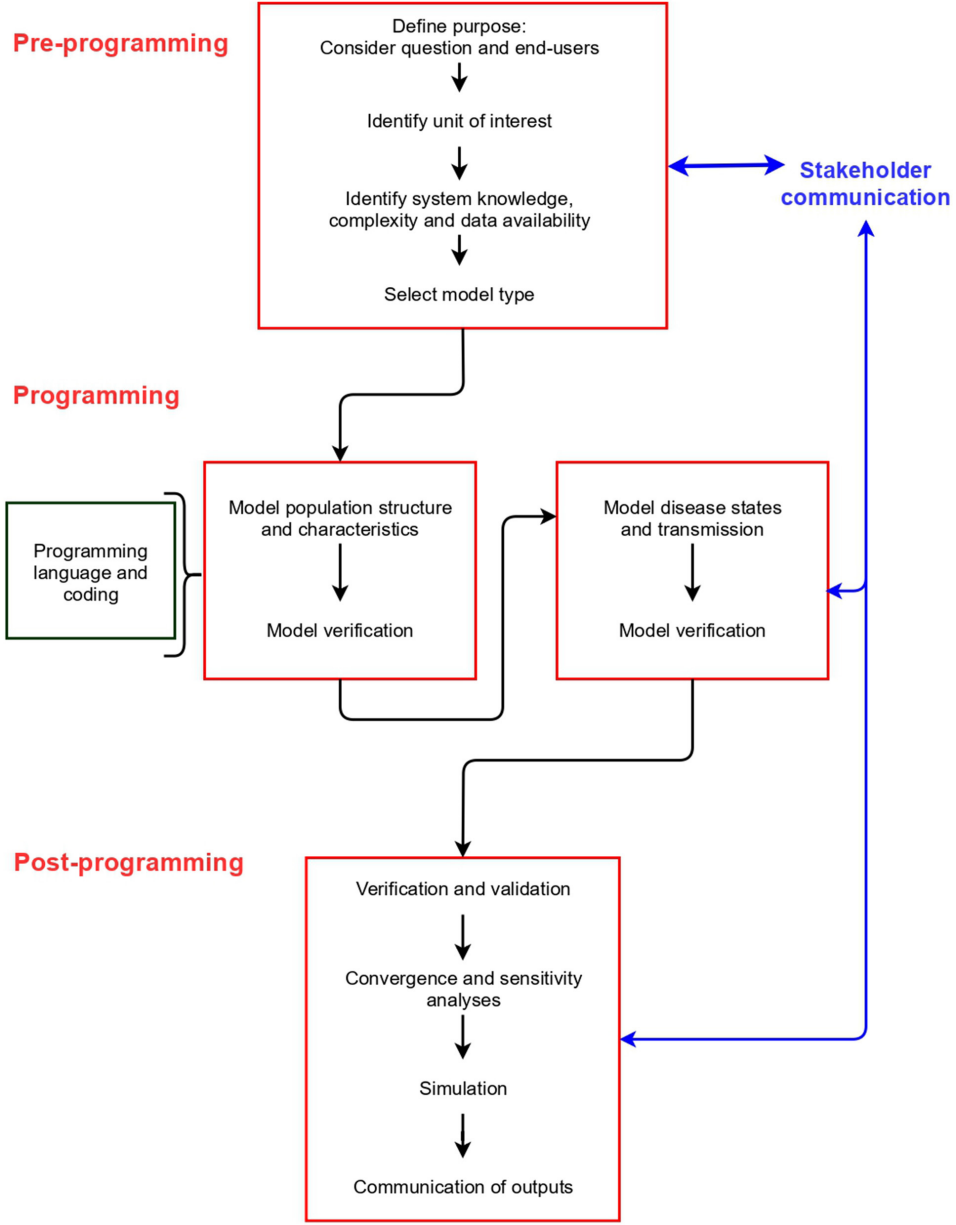
Stages, and steps within each stage, in building a disease spread model.

Model building can be divided into three stages: pre-programming, programming and post-programming. These stages are common to all model types, and include different elements that should be considered (Figure 2). We describe the concepts associated with each stage in detail below (labeled according to Figure 2).

In Appendix 2 (and https://github.com/ckirkeby/MDT), code examples are shown. We include code for a *difference equation model*, and a *differential equation model* (two model types not addressed in this article, but to enable the readers to compare the inputs and outputs with IBMs), and IBMs, for which we include examples of an *individual-based stochastic model (at herd level)*, and an *individual-based stochastic model (at individual animal level)*. We link the code for IBMs with each stage below.

#### Pre-programming Stage

##### Purpose

When designing a model, it is important to consider the research question to be investigated. This not only drives the type of model that might be appropriate, but also dictates the model outputs required by the end-user (27).

For example, whilst a model generally estimates the epidemiological consequences of the disease in terms of the number of infected individuals and epidemic duration, in the case of exotic diseases, the outputs could also be needed for contingency planning to improve surveillance and control; for example, identifying sentinel herds, culling capacity, or laboratory capacity [for example, (28, 29)]. In this case, it is essential to generate capacity-related data, such as the number of surveillance teams required, by including these parameters in the model. Similarly, if the purpose is to compare different surveillance strategies, sensitivity and specificity of tests used to detect disease need to be included (30, 31).

Evaluation and identification of optimal control strategies given a particular set of circumstances and constraints might also be a goal [for example (12, 17, 23, 32)]. This would require policy-specific knowledge to inform model processes, as well as data and knowledge of mechanisms to simulate control strategies. For example, to simulate vaccination, estimates of vaccination-specific parameters such as the number of individuals or herds vaccinated per day, vaccine efficacy, time required to order vaccine and perform vaccination could be included (9, 32). In addition to epidemiological metrics, the optimal control strategies could be defined according to economic outputs (33) such as in a bio-economic disease spread model [for example, (11)].

In the context of an IBM, the minimum inputs that must be included are a parameter to describe disease transmission (β; see later), and the number of individuals in each disease state. This will include at least one infectious individual as well as susceptible individuals (see code example, Appendix 2; https://github.com/ckirkeby/MDT). Additional parameters, such as the number of surveillance teams deployed, can be included as the model steps through the discrete time intervals; for example, in response to trigger levels such as a threshold number of infected animals for disease detection.

##### Unit of Interest

The largest unit of interest is selected so the disease spread model sufficiently represents the true system. As described previously, this epidemiological unit of the model can range from individuals [e.g., (16)] or their parts [e.g., (12)] to sub- or entire populations (34).

The choice of epidemiologic unit of interest is highly dependent on the purpose of the model, the disease of concern and the data available to parameterize the model. In models in which disease spread needs to be captured at the individual animal level (for example, because disease detection or control is performed at this level), individual animals are modeled and followed. In the case of modeling the spread of an exotic disease in animals aggregated in herds, the herd might be a more realistic unit to model, because surveillance and decisions occur at the herd-level.

Practical programming considerations also influence the choice of this unit of interest. For example, it is more likely that individual animals as units of interest are computationally more challenging, and therefore, herds are often mire suitable to be the epidemiologic unit of interest (see also Section *Programming stage*). In some systems, there might be more than one unit of interest to be modeled, as in the case of vector-borne diseases—both the vector and the animal can be units of interest (35).

In Appendix 2 we provide code examples of IBMs using different units of interest (also available online at https://github.com/ckirkeby/MDT).

##### System Knowledge, Complexity, and Data Availability

To create a model that is a sufficient representation of a real-life system, decisions need to be made about which known processes to include and exclude. This decision is bound to available information on the system. Such information is important to gather prior to model building to assess the level of uncertainty that is due to limited knowledge, how much data about the system is available, and the feasibility of delivering requested outputs. If essential data are missing to fulfill the designated purpose, options include collecting more data before modeling is initiated, re-specifying model complexity, or re-evaluating the model purpose. Following the principle of parsimony, a model should only be as complex as necessary to achieve the model purpose, thereby requiring the minimum number of assumptions (36).

Processes that should be considered include the population dynamics of the unit of interest (birth and death rate, and lifespan—this is usually based on age, or in the case of a livestock production system, this could be parity), migration of individual units in and out of the system, the contact patterns between the units and the production system of the modeled population (for example, milk or beef production), if this is relevant. It also includes knowledge of the epidemiology of the disease to be modeled, such as the relevant disease states and their durations, the modes of transmission of the causative pathogen (for example, whether or not airborne spread is an essential pathway of transmission) and how the disease develops in the individuals.

##### Model Type Selection

Model specification (units of interest, disease, and system dynamics and how they are modeled—for example, discrete vs. continuous time and deterministic vs. stochastic) is typically an iterative process and is re-examined as data gathering for parameterization occurs (Figure 2, section Documentation and Communication). If data about population dynamics, disease dynamics and the system in which disease occurs are available at an individual level, and modeling at this level of detail and heterogeneity is considered valuable (for example, if the population is small or heterogeneity of the system is considered an important feature of disease transmission), an IBM is likely suitable. Otherwise, other model types can be considered (4).

**Figure 3:**
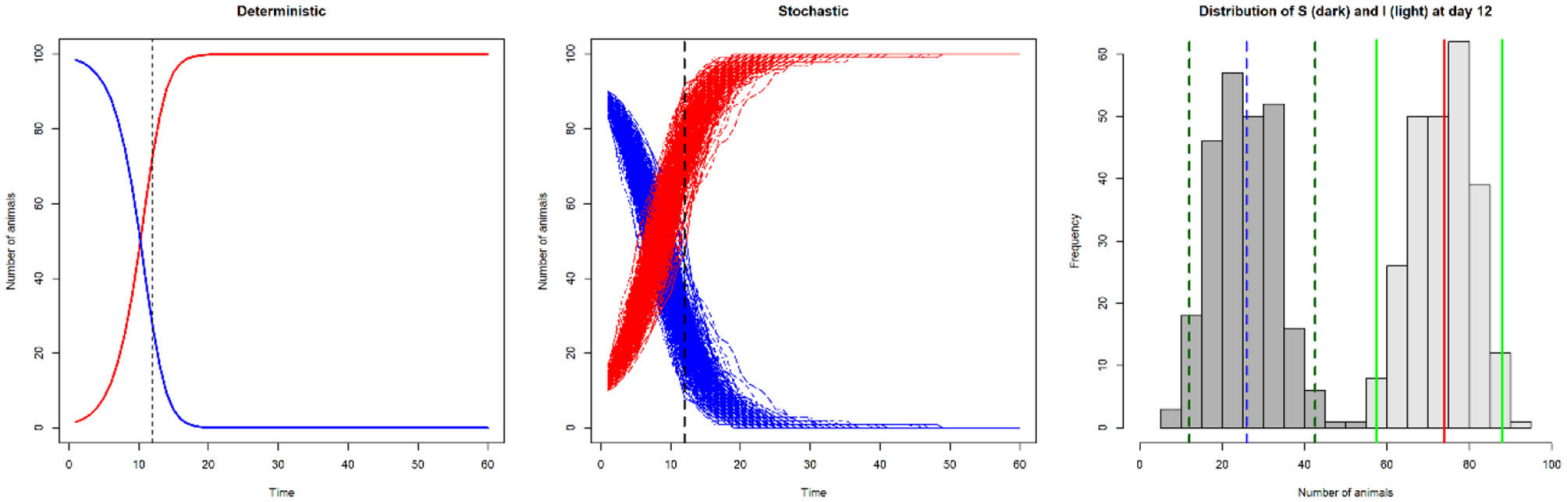
Line plots of number of susceptible (S; blue) and infected (I; red) animals in a model with SI disease dynamics, illustrating deterministic output (left: number of infected and susceptible animals on day 12 is 73 and 27, respectively), and stochastic output (centre). The histogram illustrates the variation in the number of susceptible (dark gray; median 26 [blue dashed line], 95% CI 11–42 [dark green dashed lines]) and infected animals (light gray; median 74 [red line], 95% CI 58–89 [light green lines]) from the stochastic output at day 12.

In Figure 3 we show the difference in output between a deterministic and a stochastic model.

#### Programming Stage

##### Programming Language and Coding

Programming languages can be classified in many ways—such as whether interpreted directly or compiled (running one single line of code at a time, rather than all the code has to be run together; for example, Python and R vs. C++ and Fortran, respectively); and whether they are “high” or “low” level languages. This latter classification refers to the machine-readability of the language; many languages used in the context of disease modeling can be considered high-level (for example, Java, C++, R, and Python).

In general, programs written using high-level languages require more memory space but are more readable by a human, and therefore more accessible to people without detailed programming knowledge. Programs written using low-level languages (e.g., Assembly language) can better utilize hardware specific features. These programs require a high level of knowledge to write and maintain. They can be hardware-dependent making them less portable between computer architectures.

Features resulting from language classification are not always exclusive; with many factors affecting the overall performance and efficiency of a program. For example, a complex “real-world” program written in a more user-friendly and high-level language with a modern optimizing compiler can produce highly efficient machine code with excellent performance. The result is likely to outperform an equivalent program hand-written in the less user-friendly, low-level Assembly language converted to machine code via an assembler. Advances in computational power and improvements in system architecture enable the horizontal scaling of models by running processes in parallel across multiple cores to reduce “wall time” (the time taken to complete a simulation).

Focusing on final run speed also ignores the concept of overall programming productivity. Programming in some languages is more challenging and less accessible to the research team, which increases the time required for programming. An increasing number of researchers use the free software R (37), which is a statistical programming language suitable for building many model types, including equation-based [for example, (38)] and individual-based models [for example, (11, 32)]. There are many packages available for languages such as R, and they are well-supported and maintained by R's open-source community, which allows the team to focus on modeling the system and the disease.

In regards to code programming, we highly recommend that modelers annotate their code during modeling with detailed descriptions of each part of the code. For a description of good practice in animal health modeling, see EFSA (39). Annotation assists the modeler to remember the function of each line of code, and also facilitates use of the model by others. Following publication of a study, it is a requirement of many journals that the code be made available to readers. Version control such as git (https://git-scm.com, accessed 10/09/2019) is a very valuable tool so that modelers can easily track changes in the code, and view previous versions (branches) of the model. This is of particular value when more than one modeler is involved in the project or when published code is used by other researchers. Locally, version control can be as simple as saving the script in a new file named with the specific day it is changed. We also highly recommend that during the programming process, each line or chunk of co-de should be executed with fictitious inputs to check for errors (debugging). This is part of the model verification (see section *Model Verification and Validation* for more details).

##### Modeling the Population Structure and Characteristics

Initially when constructing an IBM, the host population dynamics are modeled as the “background” for the disease dynamics. For example, a model of canine rabies spread requires a population of dogs or a foot-and-mouth disease model the population of cloven-hoofed animals. An understanding of the population of interest's demographics are critical. Whilst demographic data for livestock populations can often be gained from government or industry sources, it might be necessary to conduct studies of other populations (such as companion animals) prior to modeling to for example determine age structure and birth and death rates (40).

The population dynamics are linked to the disease model; for example, newborns can be susceptible, infected or immune (see section *Modeling disease transmission*). Also, characteristics can be allocated to the units of interest in case they influence disease transmission. In an example of Johne's disease (paratuberculosis) transmission, individual cattle or herds are modeled, and characteristics, such as individuals' milk production and lactation duration, are included because these characteristics influence disease spread [e.g., (11, 41)].

In disease spread models, it can be important to include a spatial component to the population to allow spatio-temporal modeling of disease transmission (see section *Modeling disease transmission*). This can be realized by using geolocations of the units of interest, e.g., farms, as a feature of the population structure [e.g., (17, 42)]. Spatio-temporal modeling could also represent population structures other than farms, as in the case of modeling spatio-temporal distributions of vectors that transmit bluetongue virus (43), or in the location of dog's residence in a rabies transmission model (44).

Once the background structure of the disease dynamic system has been modeled, it should be verified and tested (see sections *Model verification and validation*) before disease transmission is added to the model. This is to ensure that the model simulates the system with sufficient accuracy, as well as to determine computing requirements such as the number of iterations required for burn-in (see section *Modeling disease transmission*).

##### Modeling Disease States

As discussed previously, each stage of disease in the transmission model should reflect a -state during the course of infection in the modeled system. In the simplest framework, an *SI* model with two, mutually exclusive disease states; *Susceptible (S)* and *Infectious (I)*, all individuals in the model are assigned to either *S* or *I* (see code examples in Appendix 2; https://github.com/ckirkeby/MDT). For each simulated time step, each individual has a probability of acquiring infection and thus transitioning from *S* to *I*, depending on the contact pattern between individuals and the disease transmission rate given a contact. In the case of the *SI* model, there is no probability of individuals returning to the *S* state. In the case that animals can recover from the disease, the model becomes an *SIS* model in which infectious individuals return to the *S* state. The transmission from *I* to *S* is quantified by the recovery rate (see below, in the context of an SIR model), which can be influenced by self-recovery or by treatment. The recovery rate is thus a probability of recovering during each time step. Recovery rates must be estimated from epidemiological studies on the duration of infection. This duration of infection can either be modeled as a fixed timespan, i.e., a fixed number of days can be assigned to it, or as a distribution, after which it will revert to the *S* state.

Another common framework is the SIR model (see the code example in Appendix 2, https://github.com/ckirkeby/MDT), in which the infectious individuals can enter the *Recovered* (*R*) state – which represents either “recovery” (and resistance to infection) or “removal” from the population; for example, in the case of a rabies model, infected dogs always die and therefore are removed. The transition from *I* to *R* is also modeled via a recovery rate (denoted as “r” in the code example). Following this logic, the disease transmission framework can be further extended dependent on the disease; for example, by introducing an *Exposed (E)* state for latently infected individuals before progressing to the *I* state. As previously mentioned, even if some disease states occur in reality, it is not always useful or necessary to represent them in the model.

In the case of modeling endemic diseases, once the population and disease dynamics frameworks are modeled, an IBM might need to be simulated for enough time steps to reach a stable prevalence (“burn-in” period; the number of time steps for the population characteristics and the disease prevalence to stabilize). When such a model is used to assess control strategies, these strategies are usually implemented after the burn-in period, when a stable situation has been reached.

##### Modeling Disease Transmission

The process of disease transmission is the core dynamic process in the model. Generally, transmission can be considered as either direct (from host to host) or indirect, for example via the environment or vector transmitted (45). It can also be dependent on model features that increase contact heterogeneity; for example, some models are spatially explicit and the probability of transmission varies according to distance, mimicking a system in which transmission varies with spatial location (46).

Since disease transmission is the core process in a disease transmission model, we guide the reader through the foundation of this in the context of an IBM, such as those shown in code in Appendix 2 (https://github.com/ckirkeby/MDT). In the case of direct transmission, we first describe β, a parameter that underpins the modeling of disease transmission in equation based models, and then we describe how this parameter can be used in IBMs (47). Beta is defined as the per capita rate at which two specific individuals come into effective contact per unit time [sometimes called the transmission rate; Vynnycky and White (48)]. An effective contact is one which is sufficient for disease transmission to occur. This effective contact rate, β, comprises a contact rate between individuals (*C*), and the probability of transmission per contact (*P*):


(1) $$\begin{array}{l} \beta =C\cdot P \end{array}$$

The contact rate C in the above equation is defined per unit time, and is fundamentally different between density-dependent or frequency-dependent transmitted diseases (49–51). In density-dependent transmission, the greater the density of individuals, the greater the probability of contact per unit time (52):


(2) $$\begin{array}{l} \frac{dI}{dt}=\;\beta \cdot S\cdot I \end{array}$$

where *dI/dt* is the rate of new infections per unit time *t*, β is the effective contact rate, and *S* and *I* are the number of susceptible and infected individuals, respectively.

In frequency-dependent transmission, the rate of new infections per unit time, *dI/dt*, is independent of the density of individuals in the population (*N*):


(3) $$\begin{array}{l} \frac{dI}{dt}=\beta^{\prime }\cdot \frac{S\cdot I}{N} \end{array}$$

where *S* and *I* are the same as in Equation 2, but β′ is not equivalent to β in Equation 2 due to the underlying difference between the contact rates (*C*) of these two types of transmission. The difference between these two types of transmission is demonstrated in a study of mange in a fox population in the UK, in which researchers compared density and frequency dependent transmission and found that mange transmission was most likely frequency dependent in this population (53).

As an example of a method to allow a random process of becoming infected that can be used at each time step in an IBM, we extend Equation 3 to calculate a probability of infection per susceptible individual, *P(S)*, so each individual can be separately subjected to a Bernoulli process of becoming infected (54):


(4) $$\begin{array}{l} P\left(S\right)=1-\text{exp}\left(-\beta^{\prime }\cdot \frac{I}{N}\right) \end{array}$$

with the same notation as for Equation 2, and *N* is the total number of individuals in the modeled population. If β is fixed, then the probability of infection for all susceptible individuals is the same (for all individuals and all simulated time), and assumes homogeneity of transmission in the population. In IBMs, β may vary from one individual to another representing the susceptibility and infectiousness of the individual, thus representing natural heterogeneity in transmission. This could be driven by a lower probability of infection as a result of, for instance, vaccination or due to different contact rates between individuals.

The R code examples demonstrate this type of transmission in Appendix 2 (https://github.com/ckirkeby/MDT). In this way, the infection pressure is scaled to the proportion of the population that are infected within each time step, i.e., *I* changes over time, whereas β and *N* (within a closed system) remain constant. The infection process is dynamic because the *P(S)* changes over time with changing numbers of *I* in the population (assuming a fixed *N* and β).

As mentioned at the start of this section, it is possible to consider the spatial structure of the underlying demography and define the probability of effective contact per time step for a susceptible unit of interest dependent on its distance from infectious units in the model. For this approach, distance kernels can be built from which the probability of effective contact can be drawn (such as used in 8, 23). This spatially dependent contact rate can be combined with information on the frequency of contacts between units of interest. For example, the frequency of potential contacts between herds may not only depend on the distance between them, but also on the frequency of movements between herds, which in turn may depend on the herd types (55, 56).

When appropriate knowledge and data are available, the contact structure of a population can be based on a social network (18, 57). A heterogeneous herd contact structure between groups of animals (for example, calves and heifers) and homogenous contacts within animal groups might also be described (11, 12).

There are also several ways to simulate indirect (environmental) disease transmission. It can be similarly spatially dependent as described for the direct transmission, or simulated as a fixed transmission probability:


(5) $$\begin{array}{l} P\left(S\right)=1-\text{exp}\left(-\beta_{i}\right) \end{array}$$

Here, *P(S)* is the probability of infection of a susceptible individual *S*, and β_*i*_ is the indirect disease transmission rate. This fixed transmission rate can be based on a stable baseline infection pressure, or more variable, such as bacteria from infected individuals shed over time in the environment (11).

When disease transmission occurs through both direct and indirect contacts, a combination of both of these direct and indirect pathways can be used (12).

**Figure 4:**
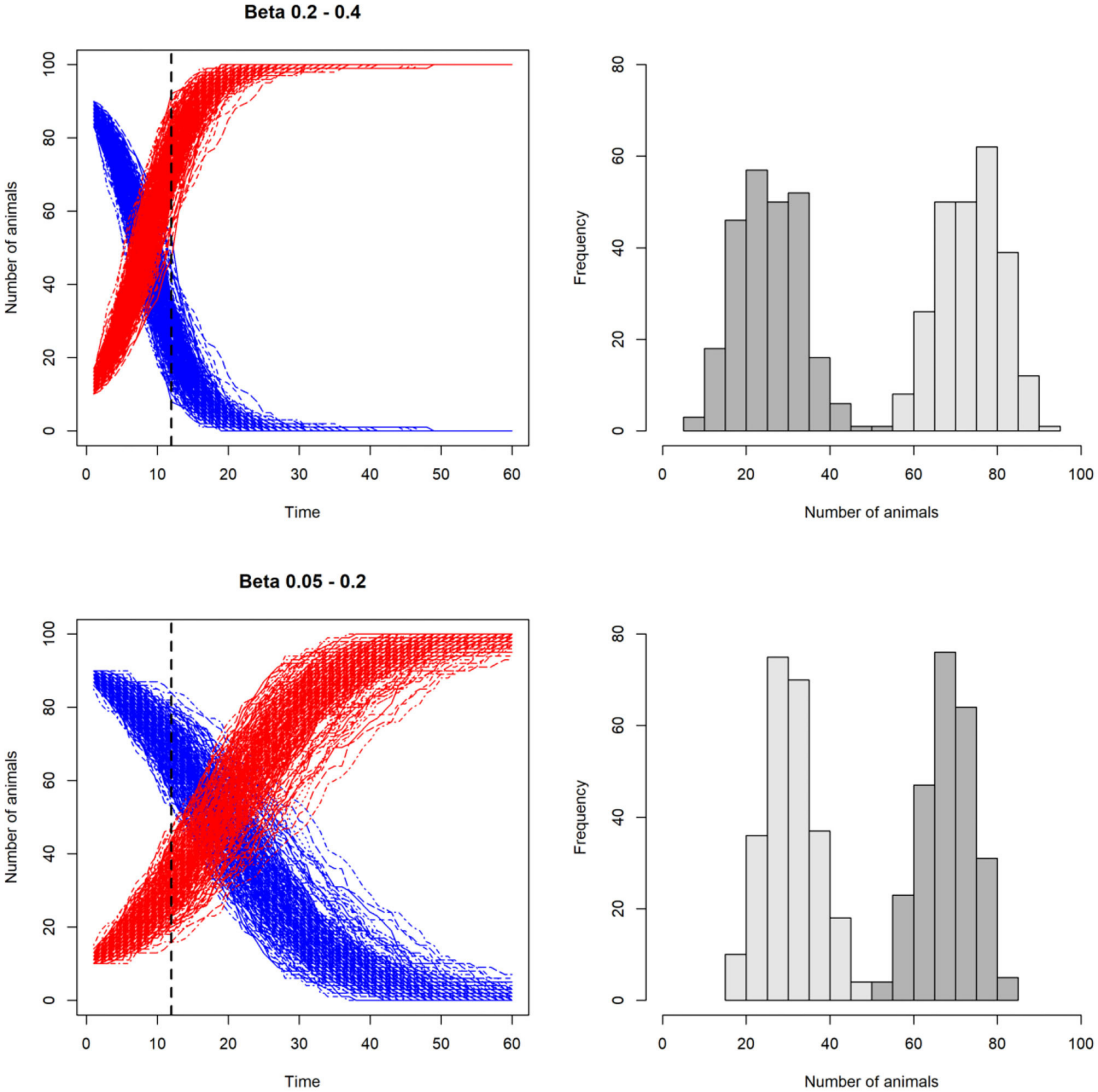
Line plots illustrating the effect of varying the transmission rate, β, on the number of susceptible (*S*) and infected (*I*) animals in a stochastic model with SI disease dynamics. In the two upper plots, β is higher than the two lower plots. This results in higher number of infected than susceptible at day 12 in the upper scenario compared to the lower scenario. In the histograms on the right, the resulting distributions of susceptible (dark gray) and infected (light gray) individuals are shown. Note that β is not kept constant, but varied for each iteration, incorporating uncertainty around this key parameter.

In Figure 4 we show an example of an SI model in which the transmission rate, β, is varied.

#### Post-programming Stage

##### Model Verification and Validation

Model verification and validation is essential to ensure that model concepts, programming and outputs are reliable, accurate, and representative for the modeled system (27, 58). Model verification ensures that model code and the conceptual framework are implemented correctly. Verification is also called computerized model verification, internal validation, or conceptual validation (58). Several methods can be used for model verification, including: (1) The rationalism method, in which several scenarios are simulated with different inputs, and outputs are compared to determine whether the changes in outputs are rational given the changes in the inputs (sensitivity analysis, see below); (2) The tracing method, in which individuals or other units of interest are followed through the different time steps and checked that they behave as expected; and (3) The face validitation method, in which an expert is asked to evaluate the outputs or even the code to verify the credibility of the model.

Model validation (also called external or operational validation) ensures that the model predictions have a satisfactory range of accuracy in relation to the actual behavior of the modeled system in real life (adapted from 54). Real-life data (i.e., empirical outbreak data) is needed to fully execute this process. To our knowledge, few models in veterinary science have been externally validated (59–61). This is usually due to the high associated costs or ethics of obtaining such data, and the complexity of the modeled systems. If empirical outbreak data are lacking from the setting in which the model was built and applied—such as in the case of exotic diseases and regions with historical disease freedom—then validation options might include either adapting the model to a region where data are available, or using previous outbreak data. For example, historical data from the last Swiss FMD outbreak was used to validate a current FMD model for Switzerland (61).

##### Convergence Analysis

Convergence analysis assesses the repeatability of the outputs based on the number of iterations (repetitions) the model is simulated, and is conducted before final model simulations. Above a given threshold of simulations, the output statistics should be independent of the number of model iterations. This stability can be checked by ensuring that the variance of the outputs of interest (for example, the number of infected individuals or epidemic duration) is stable. A commonly used approach is to visualize the change in the variance when increasing the number of iterations (62), or to use thresholds of the coefficient of variance as a decision metric (9, 18, 63).

We have included an example of how to determine convergence of a model in Appendix 2 (https://github.com/ckirkeby/MDT).

##### Sensitivity Analysis

Sensitivity analysis is essential to understand and examine the robustness of model predictions to changes in input parameter values, model structure and processes (64). Sensitivity analysis can be used to identify parameters and processes that have a major influence on model predictions; therefore, the values of these parameters—and the way in which processes are modeled—must be certain enough to produce model predictions acceptable to the end-user.

During sensitivity analysis, the behavior of the model and the outputs of interest are examined when the model or its parameters are varied. There are different ways to approach sensitivity analysis. Sensitivity analysis of input parameters can be assessed by changing input values within a specified range (local sensitivity analysis) or the entire parameter space (global sensitivity analysis) to examine the impact of these changes on model outputs. The influence of parameters can also be examined singly (one-at-a-time sensitivity analysis) or in combination with other parameters [for example, a “Sobol” sensitivity analysis, (65)]. Sensitivity analysis can also be implemented by modeling a specific process in alternative ways to examine the impact of this process on model predictions (this is sometimes referred to as structural sensitivity analysis).

The simplest method of sensitivity analysis of input parameters is one-at-a-time perturbations (66). However, this does not allow assessment of the sensitivity of the model output to changes in combinations of other parameter values' change. Many more methods exist and have been used in the context of IBMs (10, 66, 67); a complete review is beyond the scope of this article.

We have included code in Appendix 2 to conduct a simple sensitivity analysis on a model parameter (also available online at https://github.com/ckirkeby/MDT).

##### Presentation of Model Outputs

Presentation of clear results that deliver project requirements is an important element for transparent communication of the model outputs. This should already be reflected and incorporated during the design stage. Deterministic models provide single value outputs (without variation), whereas stochastic models provide distributions of outputs. Thus, when results from stochastic models are presented, it is essential to not only show median or mean values, but also the variation around these values; for example, using boxplots or histograms. From a disease spread model, outputs usually include the number of infected units of interest and the epidemic duration. Other outputs can also include the number of units of interest under control (culled, vaccinated, or banned in movements), economic metrics in case of a bio-economic model, predicted changes in production (such as milk yield or growth rates), or maps from spatially-explicit models.

#### Documentation and Communication

Good documentation is essential to enable reproducibility of the model, communication of model outcomes, and comparison between different models. Standardized protocols for disease spread model documentation have been developed, such as the ODD (Overview, Design concepts, and Details) (68) and TRACE (69) and can be used to communicate models in scientific publications.

At all stages of model design, development and implementation, communication should be maintained with relevant stakeholders. These will include the end-users of the model, but can also include experts for the specific disease and system modeled, and those that are funding model development and implementation. Comprehensive communication at all stages ensures that the model focus remains on the defined purpose so that useful information is provided to the end-users, or that the end-user can adapt the model according to specific needs during the modeling process.

#### Recent Developments

Recent developments in disease spread models used in veterinary science include the development of models that model more than one disease. Mostert et al. (70) present a bio-economic stochastic dynamic model that simulates subclinical and clinical ketosis, mastitis, metritis, displaced abomasum, and lameness in dairy cattle. In intense production systems, such as in the dairy sector, it is an advantage to evaluate the impact of several diseases concurrently, to optimize management strategies. Inclusion of economic impacts and the economics of disease mitigation in these models facilitates broader use, in addition to improving animal welfare.

Many populations can also be captured in one model. One example is the trend for models of vector-borne diseases (which we have not covered here, and introduces at least one more population, the vector, into the model).

Ensemble modeling is a relatively new approach in veterinary epidemiology (71). Decisions on how to respond to an incursion of FMD virus in a previously disease-free country are complex and several models of FMD spread have been developed and applied. These vary in their disease processes modeled, assumptions made and parameterization. For any set of inputs, outputs from these various models are plausible. Variability in model outputs can be valuable because these are likely to include the range of realizations that could be observed during an FMD outbreak. A method of reconciling variability—borrowed from fields such as meteorology, climate-change science and medical science—has recently been applied to this situation. Using outputs from six different models which simulated the spread of FMD in the Midlands and Wales areas of the United Kingdom in 2001, Webb et al. (71) applied a Bayesian Reliability Ensemble Average (BREA) method to integrate outputs regarding outbreak duration and two control methods. The BREA method determines the weights applied to each model output based on agreement with observed data (bias criterion) and consensus between models (convergence criterion). The latter was used by Webb et al. (71) and their case study highlights the potential of ensemble modeling to reduce the uncertainty of outputs from individual models, thus improving decision-making.

## Conclusions and Recommendations

We emphasize two well-known, key axioms: (1). disease spread models are simplified representations of real-life systems so that “all models are wrong, but some are useful” (2), and (2). model outputs can only be as accurate as model inputs allow.

Model simplification is often driven by data availability; therefore, full use of any available data is recommended. However, when considering whether more data should be collected or how a process should be modeled, we note that highly detailed models (more complex processes with more parameters, such as IBMs) can produce output that might be less generalizable than more simplified models. In addition, the output from more simplified models might adequately predict the essential components of disease transmission needed to achieve the end-users' objectives. This presents modelers with dilemmas: a highly detailed model is not necessarily less “wrong” or more “useful” than a simplified model. Whilst the steps of model verification, validation, and sensitivity analysis can help avoid too much or too little simplification, we recommend that particularly during the design phase, modelers focus on development of the simplest model to achieve useful output—whilst we focus on an introduction to modeling using IBMs, we do not suggest that they are the foundation of modeling approaches.

Communication between end-users and modelers about the value and assumptions of a model is critical. We therefore recommend that modelers and end-users, wherever possible, establish a framework for communication about modeling objectives, the need for verification, validation, and sensitivity analysis, and application of model outputs to ensure optimal use of simulation modeling, to improve animal health, welfare, and production.

## Data Availability Statement

The original contributions generated for the study are included in the article/Supplementary Materials, further inquiries can be directed to the corresponding author/s.

## Author Contributions

CK wrote the first draft of the manuscript. All authors participated in writing the manuscript.

## Conflict of Interest

The authors declare that the research was conducted in the absence of any commercial or financial relationships that could be construed as a potential conflict of interest.

## References

[1] Lessler J Cummings DAT. Mechanistic models of infectious disease and their impact on public health. Am J Epidem. (2016) 183:415–22. 10.1093/aje/kww021 26893297 PMC5006438

[2] Box GE. Science and statistics. J American Stat Assoc. (1976) 71:791–9. 10.1080/01621459.1976.10480949

[3] Holmdahl I Buckee C. Wrong but useful—what Covid-19 epidemiologic models can and cannot tell us. N Engl J Med. (2020) 383:303–5. 10.1056/NEJMp2016822 32412711

[4] Mancy R Brock PM Kao RR. An integrated framework for process-driven model construction in disease ecology and animal health. F Vet Sci. (2017) 4:155. 10.3389/fvets.2017.00155 29021983 PMC5623672

[5] Singer A Salman M Thulke HH. Reviewing model application to support animal health decision making. Prev Vet Med. (2011) 99:60–7. 10.1016/j.prevetmed.2011.01.004 21306779

[6] Zinsstag J Dürr S Penny MA Mindekem R Roth F Gonzalez SM . Transmission dynamics and economics of rabies control in dogs and humans in an African city. PNAS. (2009) 106:14996–5001. 10.1073/pnas.0904740106 19706492 PMC2728111

[7] Thulke H-H Lange M Tratalos JA Clegg TA McGrath G O'Grady L . Eradicating BVD, reviewing Irish programme data and model predictions to support prospective decision making. Prev Vet Med. (2018) 150:151–61. 10.1016/j.prevetmed.2017.11.017 29221591

[8] Halasa T Boklund A Bøtner A Mortensen S Kjær LJ. Simulation of transmission and persistence of African swine fever in wild boar in Denmark. Prev Vet Med. (2019) 167:68–79. 10.1016/j.prevetmed.2019.03.028 31027724

[9] Dürr S Ward MP. Development of a novel rabies simulation model for application in a non-endemic environment. PLoS Negl Trop Dis. (2015) 9:e0003876. 10.1371/journal.pntd.0003876 26114762 PMC4482682

[10] Brookes VJ Jordan D Davis S Ward MP Heller J. Saltelli global sensitivity analysis and simulation modelling to identify intervention strategies to reduce the prevalence of *Escherichia coli* o157 contaminated beef carcasses. PLoS ONE. (2015) 10:e0146016. 10.1371/journal.pone.0146016 26713610 PMC4694618

[11] Kirkeby C Græsbøll K Nielsen SS Christiansen LE Toft N Rattenborg E . Simulating the epidemiological and economic impact of paratuberculosis control actions in dairy cattle. Fron Vet Sci. (2016) 3:90. 10.3389/fvets.2016.00090 27777933 PMC5056316

[12] Gussmann M Kirkeby C Græsbøll K Farre M Halasa T. A strain-, cow-, and herd-specific bio-economic simulation model of intramammary infections in dairy cattle herds. J Theor Biol. (2018) 449:83–93. 10.1016/j.jtbi.2018.04.022 29678690

[13] Zingg D Steinbach S Kuhlgatz C Rediger M Schüpbach-Regula G Aepli M . Epidemiological and economic evaluation of alternative on-farm management scenarios for ovine footrot in Switzerland. Front Vet Sci. (2017) 4:1–13. 10.3389/fvets.2017.00070 28560223 PMC5432651

[14] Kudahl AB Østergaard S Sørensen JT Nielsen SS. A stochastic model simulating paratuberculosis in a dairy herd. Prev Vet Med. (2007) 78:97–117. 10.1016/j.prevetmed.2006.05.015 17112610

[15] Halasa T Nielen M van Werven T Hogeveen H. A simulation model to calculate costs and benefits of dry period interventions in dairy cattle. Livestock Sci. (2010) 129:80–7. 10.1016/j.livsci.2010.01.009

[16] Groenendaal H Nielen M Jalvingh AW Horst SH Galligan DT Hesselink JW. A simulation of Johne's disease control. Prev Vet Med. (2002) 54:225–45. 10.1016/S0167-5877(02)00027-2 12114011

[17] Backer JA Hagenaars TJ Nodelijk G van Roermund HJW. Vaccination against foot-and-mouth disease I: epidemiological consequences. Prev Vet Med. (2012) 107:27–40. 10.1016/j.prevetmed.2012.05.012 22749763

[18] Brookes VJ Dürr S Ward MP. Rabies-induced behavioural changes are key to rabies persistence in dog populations: investigation using a network-based model. PLoS Negl Trop Dis. (2019) 13:e0007739. 10.1371/journal.pntd.0007739 31545810 PMC6776358

[19] Laager M Mbilo C Madaye E. A Naminou A Léchenne M Tschopp A . The importance of dog population contact network structures in rabies transmission. PLOS Negl Trop Dis. (2018) 12:e0006680. 10.1371/journal.pntd.0006680 30067733 PMC6089439

[20] Kermack WO McKendrick AG. A contribution to the mathematical theory of epidemics. Proce R Soc London Series A. (1927) 115:700–21. 10.1098/rspa.1927.0118

[21] Mahsin M Deardon R Brown P. Geographically-dependent individual-level models for infectious diseases transmission. Biostatistics, 21. 10.1093/biostatistics/kxaa009 32118253

[22] Mardones F Perez A Sanchez J Alkhamis M Carpenter T. Parameterization of the duration of infection stages of serotype O foot-and-mouth disease virus: an analytical review and meta-analysis with application to simulation models. Vet Res. (2010) 41:45. 10.1051/vetres/2010017 20205988 PMC2850150

[23] Ward MP Highfield LD Vongseng P Garner MG. Simulation of foot-and-mouth disease spread within an integrated livestock system in Texas, USA. Prev Vet Med. (2009) 88:286–97. 10.1016/j.prevetmed.2008.12.006 19178967

[24] Tang W Bennett DA. Agent-based modeling of animal movement: a review. Geography Compass. (2010) 4:682–700. 10.1111/j.1749-8198.2010.00337.x

[25] Hudson EG Brookes VJ Ward MP Dürr S. Using roaming behaviours of dogs to estimate contact rates: the predicted effect on rabies spread. Epidemiolo Infect. (2019) 147:e135. 10.1017/S0950268819000189 30869048 PMC6518777

[26] Mideo N Alizon S Day T. Linking within-and between-host dynamics in the evolutionary epidemiology of infectious diseases. Trends Ecol Evol. (2008) 23:511–7. 10.1016/j.tree.2008.05.009 18657880

[27] Garner MG Hamilton SA. Principles of epidemiological modelling. Rev Sci Tech. (2011) 30:407–16. 10.20506/rst.30.2.2045 21961213

[28] Garner MG Bombarderi N Cozens M Conway ML Wright T Paskin R . Estimating resource requirements to staff a response to a medium to large outbreak of foot and mouth disease in Australia. Transbound Emerg Dis. (2016) 63:e109–21. 10.1111/tbed.12239 24894407

[29] Boklund A Halasa THB Christiansen LE Willeberg P Enøe C. Simulated effects of introducing emergency vaccination or depopulation during FMD outbreaks in Denmark. In: EuFMD Meeting 2012: Open Session, Jerez (2012).

[30] Nusser SM Clark WR Otis DL Huang L. Sampling considerations for disease surveillance in wildlife populations. J Wildl Manage. (2008) 72:52–60. 10.2193/2007-317

[31] Halasa T Boklund A. The impact of resources for clinical surveillance on the control of a hypothetical foot-and-mouth disease epidemic in Denmark. PLoS ONE. (2014) 9:e102480. 10.1371/journal.pone.0102480 25014351 PMC4094525

[32] Dürr S Fasel-Clemenz C Thür B Schwermer H Perler L Doherr MG . Evaluation of the benefit of emergency vaccination in a foot-and-mouth disease free country with low livestock density. Prev Vet Med. (2014) 113:34–46. 10.1016/j.prevetmed.2013.10.015 24211105

[33] Gethmann J Probst C Bassett J Blunk P Hövel P Conraths FJ. An epidemiological and economic simulation model to evaluate strategies for the control of bovine virus diarrhea in Germany. Front Vet Sci. (2019) 6:406. 10.3389/fvets.2019.00406 31803768 PMC6877714

[34] Kopec JA Finès P Manuel DG Buckeridge DL Flanagan WM Oderkirk J . A stochastic model simulating paratuberculosis in a dairy herd. Prev Vet Med. (2007) 78” 97–117. 17112610 10.1016/j.prevetmed.2006.05.015

[35] Græsbøll K Bødker R Enøe C Christiansen LE. Simulating spread of bluetongue virus by flying vectors between hosts on pasture. Sci Rep. (2012) 2:863. 10.1038/srep00863 23162689 PMC3499760

[36] Lilien GL. Model relativism: a situational approach to model building. Interfaces. (1975) 5:11–18. 10.1287/inte.5.3.11

[37] R Core Team. R: A Language and Environment for Statistical Computing, version 3.3. 1. Vienna: R Foundation for Statistical Computing; 2016 (2019).

[38] Frasso G Lambert P. Bayesian inference in an extended SEIR model with nonparametric disease transmission rate: an application to the Ebola epidemic in Sierra Leone. Biostatistics. (2016) 17:779–92. 10.1093/biostatistics/kxw027 27324411

[39] EFSA. Guidance on good practice in conducting scientific assessments in animal health using modelling. EFSA J. (2009) 7:209–21. 10.2903/j.efsa.2009.1419 PMC9115711 35600270

[40] Hudson EG Brookes VJ Ward MP. Demographic studies of owned dogs in the Northern Peninsula Area, Australia, to inform population and disease management strategies. Austr Veterinary J. (2018) 96:487–94. 10.1111/avj.12766 30478842

[41] Damman A Viet A-F Arnoux S Guerrier-Chatellet M-C Petit E Ezanno P. Modelling the spread of bovine viral diarrhea virus (BVDV) in a beef cattle herd and its impact on herd productivity. Vet Res. (2015) 46:12. 10.1186/s13567-015-0145-8 25828555 PMC4337316

[42] Tildesley MJ Keeling MJ. Foot-and-mouth disease - a modelling comparison between the UK and Denmark. Prevent Vetering Med. (2008) 85:107–24. 10.1016/j.prevetmed.2008.01.008 18328581

[43] Kelso JK Milne GJ. A spatial simulation model for the dispersal of the bluetongue vector Culicoides brevitarsis in Australia. PLoS ONE. (2014) 9:e104646. 10.1371/journal.pone.0104646 25105418 PMC4126746

[44] Hudson EG BrookesVJ Dürr S Ward MP. Modelling targeted rabies vaccination strategies for a domestic dog population with heterogeneous roaming patterns. PLOS Negl Trop Dis. (2019) 19:e0007582. 10.1371/journal.pntd.0007582 31283780 PMC6638970

[45] Anderson RM May RM. Infectious Diseases of Humans. New York, NY: Oxford Science Publications (1991).

[46] Mur L Sánchez-Vizcaíno JM Fernández-Carrión E Jurado C Rolesu S Feliziani F . Understanding African swine fever infection dynamics in Sardinia using a spatially explicit transmission model in domestic pig farms. Transbound Emerg. Dis. (2018) 65:123–34. 10.1111/tbed.12636 28296281

[47] Kean JM Barlow ND Hickling GJ. Evaluating potential sources of bovine tuberculosis infection in a New Zealand cattle herd. New Zealand J Agric Res. (1999) 42:101–6. 10.1080/00288233.1999.9513358

[48] Vynnycky E White R. An Introduction to Infectious Disease Modelling. Oxford University Press (2010).27866768

[49] Begon M Bennett M Bowers RG French NP Hazel SM Turner J. A clarification of transmission terms in host-microparasite models: numbers, densities and areas. Epidemiol Infect. (2002) 129:147–53. 10.1017/S0950268802007148 12211582 PMC2869860

[50] Ryder JJ Miller MR White A Knell RJ Boots M. Host-parasite population dynamics under combined frequency-and density-dependent transmission. Oikos. (2007) 116:2017–26. 10.1111/j.2007.0030-1299.15863.x

[51] Keeling MJ Rohani P. Modeling Infectious Diseases in Humans and Animals. Princeton, NJ; Oxford: Princeton University Press, (2008). 10.2307/j.ctvcm4gk0

[52] McCallum H Barlow N Hone J. How should pathogen transmission be modeled? Trends Ecol Evol. (2001) 16:295–300. 10.1016/S0169-5347(01)02144-9 11369107

[53] Devenish-Nelson ES Richards SA Harris S Soulsbury C Stephens PA. Demonstrating frequency-dependent transmission of sarcoptic mange in red foxes. Biol Lett. (2014) 10:20140524. 10.1098/rsbl.2014.0524 25296930 PMC4272203

[54] Kretzschmar M Wallinga J. Mathematical models in infectious disease epidemiology. In: Mirjam Kretzschmar, editor. Modern Infectious Disease Epidemiology. New York, NY: Springer (2009). p. 209–21. 10.1007/978-0-387-93835-6_12

[55] Bates TW Thurmond MC Carpenter TE. Results of epidemic simulation modeling to evaluate strategies to control an outbreak of foot-and-mouth disease. Am J Vet Res. (2003) 64:205–10. 10.2460/ajvr.2003.64.205 12602590

[56] Ferguson NM Donnelly CA Anderson RM. The foot-and-mouth epidemic in Great Britain: pattern of spread and impact of interventions. Science. (2001) 292:1155–60. 10.1126/science.1061020 11303090

[57] Hirsch BT Reynolds JJ Gehrt SD Craft ME. Which mechanisms drive seasonal rabies outbreaks in raccoons? A test using dynamic social network models*. J Appl Ecol*. (2016) 53:804–13. 10.1111/1365-2664.12628

[58] Sargent RG. Verification and validation of simulation models. J Simulat. (2013) 7:12–24. 10.1057/jos.2012.20

[59] Foddai A Enøe C Krogh K Stockmarr A Halasa T. Stochastic simulation modeling to determine time to detect bovine viral diarrhea antibodies in bulk tank milk. Prev Vet Med. (2014) 117:149–59. 10.1016/j.prevetmed.2014.07.007 25081944

[60] Zinsstag J Lechenne M Laager M Mindekem R Naïssengar S Oussiguéré A . Vaccination of dogs in an African city interrupts rabies transmission and reduces human exposure. Sci Transl Med. (2017) 9:eaaf6984. 10.1126/scitranslmed.aaf6984 29263230

[61] Zingg D Häsler S Schuepbach-Regula G Schwermer H Dürr S. Evidence for emergency vaccination having played a crucial role to control the 1965/66 foot-and-mouth disease outbreak in Switzerland. Front Vet Sci. (2015) 2:72. 10.3389/fvets.2015.00072 26697436 PMC4677095

[62] Halasa T Bøtner A Mortensen S Christensen H Toft N Boklund A. Simulating the epidemiological and economic effects of an African swine fever epidemic in industrialized swine populations. Vet Microbiol. (2016) 193:7–16. 10.1016/j.vetmic.2016.08.004 27599924

[63] Cowled BD Garner MG Negus K Ward MP. Controlling disease outbreaks in wildlife using limited culling: modelling classical swine fever incursions in wild pigs in Australia. Vet Res. (2012) 43:3. 10.1186/1297-9716-43-3 22243996 PMC3311561

[64] Ezanno P Fourichon C Viet A-F Seegers H. Sensitivity analysisto identify key-parameters in modelling the spread of bovine viraldiarrhoea virus in a dairy herd. Prev Vet Med. (2007) 80:49–64. 10.1016/j.prevetmed.2007.01.005 17303270

[65] Saltelli A. Making best use of model evaluations to compute sensitivity indices. Computer Phys Commun. (2002) 145:280–97. 10.1016/S0010-4655(02)00280-1

[66] Norton J. An introduction to sensitivity assessment of simulation models. Environ Model Softw. (2015) 69:166–74. 10.1016/j.envsoft.2015.03.020

[67] Frey CH Patil SR. Identification and review of sensitivity analysis methods. Risk Analysis. (2002) 22:553–78. 10.1111/0272-4332.00039 12088234

[68] Grimm V Berger U DeAngelis DL Polhill JG Giske J Railsback SF. The ODD protocol: a review and first update. Ecol Model. (2010) 221:2760–8. 10.1016/j.ecolmodel.2010.08.019

[69] Grimm V Augusiak J Focks A Frank BM Gabsi F Johnston AS . Towards better modelling and decision support: documenting model development, testing, and analysis using TRACE. Ecol Model. (2014) 280:129–39. 10.1016/j.ecolmodel.2014.01.018

[70] Mostert PF Bokkers EAM Van Middelaar CE Hogeveen H De Boer IJM. Estimating the economic impact of subclinical ketosis in dairy cattle using a dynamic stochastic simulation model. Animal. (2018) 12:145–54. 10.1017/S1751731117001306 28637532

[71] Webb CT Carpenter TE Dürr S Ferrari M Garner MG Jewell C . Ensemble modelling and structured decision-making to support emergency disease management. Prev Vet Med. (2017) 138:124–33. 10.1016/j.prevetmed.2017.01.003 28237227

