# Corrigendum: A practical introduction to mechanistic modeling of disease transmission in veterinary science

**DOI:** 10.3389/fvets.2023.1129870

**Published:** 2023-01-23

**Authors:** Carsten Kirkeby, Victoria J. Brookes, Michael P. Ward, Salome Dürr, Tariq Halasa

**Affiliations:** ^1^Department of Veterinary and Animal Sciences, Faculty of Health and Medical Sciences, University of Copenhagen, Frederiksberg, Denmark; ^2^School of Animal and Veterinary Sciences, Faculty of Science, Charles Sturt University, Wagga, NSW, Australia; ^3^Graham Centre for Agricultural Innovation (Charles Sturt University and NSW Department of Primary Industries), Wagga, NSW, Australia; ^4^Faculty of Veterinary Science, Sydney School of Veterinary Science, University of Sydney, Sydney, NSW, Australia; ^5^Department of Clinical Research and Public Health, Veterinary Public Health Institute, University of Bern, Bern, Switzerland

**Keywords:** simulation model, transmission model, disease dynamics, mechanistic model, disease model

In the published article, there was an error. In Equation (4), incorrectly there was an “S” included on the right hand side of the equation.

A correction has been made to the section **Programming stage**, Modeling Disease Transmission, paragraph 4, Equation (4). The equation previously stated:

“P(S)=1–exp(–β′·S·I/N)”

The corrected Equation (4) appears below:

“P(S)=1–exp(–β′·I/N)”

This is because this equation shows the infection process per susceptible individual, so the “S” should not be included in the equation, as the whole expression is repeated per susceptible individual.

The authors apologize for this error and state that this does not change the scientific conclusions of the article in any way. The original article has been updated.

