# Supplementary material for: A Practical Introduction to Mechanistic Modeling of Disease Transmission in Veterinary Science
Source: Front Vet Sci. 2021 Jan 26;7:546651. doi: 10.3389/fvets.2020.546651 (PMC7870987; doi:10.3389/fvets.2020.546651)
Supplement: Supplementary file 1 [file Data_Sheet_1.docx]

APPENDIX 1

*List of key terms and their definitions used in the context of mechanistic modeling of disease transmission in veterinary science***Mapprochesmechan**

| **Term** | **Definition** |
| --- | --- |
| *ABM* | Agent-based model. A model that simulates the transmission of disease between explicit individual units of interest, and tracks the status of the individual units of interest over time. |
| *Convergence analysis* | Analysis performed to evaluate the repeatability of the results. Convergence analysis can be used to determine if a stochastic model has been run for enough iterations to cover the variation in the possible output. |
| *Deterministic model* | A model that does not include variation in the parameter values or randomness in the model processes. |
|  |  |
| *EBM* | Equation-based model. Models, such as differential or difference equation models, that do not track the individual unit of interest. |
| *IBM* | Individual-based model. A model that simulates the transmission of disease between pairs of individual units of interest, and tracks the status of the individual units of interest over time. |
| *Sensitivity analysis* | Analysis performed to evaluate the impact of parameters or processes in a model. |
| *Stochastic model* | A model that includes variation in the parameter values or randomness in the model processes. |
| *Validation* | Comparison of model results with data that were not used in the model development. This procedure evaluates the model representativeness to other situations. |
| *Verification* | Steps in the model building procedure carried out to ensure that the model performs as intended. |

APPENDIX 2

This code is also available online at <https://github.com/ckirkeby/MDT>

# Code for showing simple examples of the different types of models created in R.

#### Difference equation model example ####

beta <- 0.5 # Set transmission rate

timesteps <- 1:10 # Set time steps

S <- 9 # We start with 9 susceptble individuals

I <- 1 # And 1 infected individual

N <- S + I # The total population is S + I

n.inf <- numeric() # Create string for number of infected

n.sus <- numeric() # Create string for number of susceptible

n.pop <- numeric() # Create string for total population

for (i in timesteps)

{

dI <- beta * S * I / N # Calculate change in number of infected

I <- I + dI # Update the number of infected

S <- S - dI # Update the number of susceptible

N <- S + I # Update the total population

n.inf[i] <- I # Save the infected

n.sus[i] <- S # Save the susceptible

n.pop[i] <- I + S # Save the total population

}

# Make a plot of the simulation:

plot(timesteps, n.pop, type="b", ylab="Y", xlab="time", main="Difference equation model example", ylim=c(0,10))

points(n.inf, col="red", type="b")

points(n.sus, col="blue", type="b")

#### Differential equation model example ####

## In this type of model, a set of differential equations are usually solved in a package, such as deSolve, in R. Here we use the “ode” function to solve the equations over time.

## Load deSolve package

library(deSolve)

## Create an SIR function

SI <- function(time, state, parameters) {

with(as.list(c(state, parameters)), { # Use list of disease states and parameters

dS <- -beta * S * I # Calculate change in susceptible

dI <- beta * S * I # Calculate change in infected

return(list(c(dS, dI))) # Output the result

})

}

### Set parameters

# Set proportion infected at start:

inf.start <- 0.1

# Proportion in each compartment: 10% infected and 90% susceptible

init <- c(S = 1-inf.start, I = inf.start)

# beta: infection parameter; gamma: recovery parameter

parameters <- c(beta = 0.5)

# Time frame

timesteps <- c(1:10)

## Solve using ode (General Solver for Ordinary Differential Equations)

out <- ode(y = init, times = timesteps, func = SI, parms = parameters)

## change to data frame

out <- as.data.frame(out)

out$N <- out$S + out$I

# Make a plot of the simulation:

plot(timesteps, out$N, type="b", xlab="time",

main="Differential equation model example", ylim=c(0,1),

ylab="Proportion of population")

points(out$I, col="red", type="b")

points(out$S, col="blue", type="b")

#### Stochastic mechanistic spread model model example with population as the unit of interest####

beta <- 0.5 # transmission rate

timesteps <- 1:10 # Time steps

S <- 9 # We start with 9 susceptble individuals

I <- 1 # And 1 infected individual

N <- S + I # The total population is S + I

n.inf <- numeric() # Create string for number of infected

n.sus <- numeric() # Create string for number of susceptible

n.pop <- numeric() # Create string for total population

for(i in timesteps) # Loop over time

{

PI <- 1 - exp( - beta * I / N) # Calculate the current probability of infection

dI <- sum( rbinom(S , 1 , prob=PI) ) # Randomly assign new infections

I <- I + dI # Update number of infected individuals

S <- S - dI # Update number of susceptible individuals

N <- S + I # Update total number of individuals in population

n.inf[i] <- I # Save the infected

n.sus[i] <- S # Save the susceptible

n.pop[i] <- I + S # Save the total population

}

# Make a plot of the simulation:

plot(timesteps, n.pop, type="b", ylab="Y", xlab="time",

main="Stochastic population-based mechanistic model example", ylim=c(0,10))

points(n.inf, col="red", type="b")

points(n.sus, col="blue", type="b")

#### Stochastic mechanistic spread model model example with individuals as the unit of interest####

beta <- 0.5 # Set fixed transmission rate

timesteps <- 1:10 # Set time steps

pop <- data.frame(ID = c(1:10), inf.status = c(1 , rep(0, 9)) )

n.inf <- numeric() # Create string for number of infected

n.sus <- numeric() # Create string for number of susceptible

n.pop <- numeric() # Create string for total population

for(i in timesteps) # Loop over time

{

I <- length(pop$inf.status[pop$inf.status == 1]) # Update the infected individuals

S <- length(pop$inf.status[pop$inf.status == 0]) # Update the susceptible individuals

N <- length(pop$inf.status) # Update the total population size

PI <- 1 - exp( - beta * I / N) # Calculate the current probability of infection

new.inf <- rbinom(S , 1 , prob=PI) # Calculate which individuals will be infected

pop$inf.status[pop$inf.status == 0] <- new.inf # Assign new infections

n.inf[i] <- I # Save the infected

n.sus[i] <- S # Save the susceptible

n.pop[i] <- I + S # Save the total population

}

# Make a plot of the simulation:

plot(timesteps, n.pop, type="b", ylab="Y", xlab="time",

main="Stochastic individual-based mechanistic model example", ylim=c(0,10))

points(n.inf, col="red", type="b")

points(n.sus, col="blue", type="b")

#### Stochastic mechanistic spread model model example with individuals as the ####

#### unit of interest and a infection-dependent daily weight gain per individual ####

beta <- 0.1 # Set fixed transmission rate

timesteps <- 1:20 # Set time steps

pop <- data.frame(ID = c(1:10), inf.status = c(1 , rep(0, 9)), weight = 5 ) # Weight = 5 kg initially

daily.weight.gain <- 1 # One kg per individual.

daily.weight.reduction <- 0.9 # Reduction of daily weight gain.

n.inf <- numeric() # Create string for number of infected

n.sus <- numeric() # Create string for number of susceptible

n.pop <- numeric() # Create string for total population

n.weight <- numeric() # Create string for total population

for(i in timesteps) # Loop over time

{

I <- length(pop$inf.status[pop$inf.status == 1]) # Update the infected individuals

S <- length(pop$inf.status[pop$inf.status == 0]) # Update the susceptible individuals

N <- length(pop$inf.status) # Update the total population size

PI <- 1 - exp( - beta * I / N) # Calculate the current probability of infection

new.inf <- rbinom(S , 1 , prob=PI) # Calculate which individuals will be infected

pop$inf.status[pop$inf.status == 0] <- new.inf # Assign new infections

pop$weight <- pop$weight+1 # Calculate daily weight gain all individuals: 1 kg

pop$weight[pop$inf.status == 1] <- pop$weight[pop$inf.status == 1]-daily.weight.reduction # Reduce weight gain for the infected individuals

n.inf[i] <- I # Save the infected

n.sus[i] <- S # Save the susceptible

n.pop[i] <- I + S # Save the total population

n.weight[i] <- sum(pop$weight)

}

# Make a plot of the simulation:

text.title <- paste0("Stochastic individual-based mechanistic model example with beta = ", beta )

plot(timesteps, n.weight, type="b", ylab="Total weight of population", xlab="Time",

main=text.title)

# Try to vary beta and run the script again.

#### Stochastic individual-based mechanistic simulation model with multiple iterations example ####

MaxIterations <- 10 # Set the number of iterations to simulate

Output <- matrix(numeric(0),ncol=3) # Make a matrix for the model output with three columns: S, I and N.

timesteps <- 1:10 # Set the number of time steps

n <- 10 # population size

for(j in 1:MaxIterations) # Loop over iterations

{

beta <- runif(n,0.4,0.6) # Set interval for the transmission rate

pop <- data.frame(ID = c(1:n), inf.status = c(1 , rep(0, 9)) ) # Create the population

n.inf <- numeric() # Create string for number of infected

n.sus <- numeric() # Create string for number of susceptible

n.pop <- numeric() # Create string for total population

for(i in timesteps) # Loop over time

{

I <- length(pop$inf.status[pop$inf.status == 1]) # Update the infected individuals

S <- length(pop$inf.status[pop$inf.status == 0]) # Update the susceptible individuals

N <- length(pop$inf.status) # Update the total population size

PI <- 1 - exp( - beta * I / N) # Calculate the current probability of infection

new.inf <- rbinom(S , 1 , prob=PI) # Calculate which individuals will be infected

pop$inf.status[pop$inf.status == 0] <- new.inf # Assign new infections

n.inf[i] <- I # Save the infected

n.sus[i] <- S # Save the susceptible

n.pop[i] <- I + S # Save the total population

#

Output <- rbind(Output,c(i,sum(pop$inf.status==0),sum(pop$inf.status==1))) # Save the simulation

}

}

## If you run the model 1 iteration, you can observe the progress of the infection for that iteration using this code:

plot(timesteps, n.pop, type="b", ylab="Y", xlab="time",

main="Stochastic individual-based mechanistic model example", ylim=c(0,10))

points(n.inf, col="red", type="b")

points(n.sus, col="blue", type="b")

## If you run the model for > 1 iteration, then you can observe the effect of randomness on infection using the following code:

plot(Output[,1],Output[,2],xlab="Time", ylab="Number of animals",ylim=c(0,10),typ="l")

lines(Output[,1],Output[,3],col="red")

## To observe the progress of infection based on all iterations, median number can be ploted over time as follows:

Sus <- sapply(unique(Output[,1]),function(x) median(Output[Output[,1]==x,2]))

Infect <- sapply(unique(Output[,1]),function(x) median(Output[Output[,1]==x,3]))

plot(unique(Output[,1]),Sus,xlab="Time", ylab="Number of animals",ylim=c(0,10),typ="l")

lines(unique(Output[,1]),Infect,col="red")

#### Sensitivity analysis example ####

# Create the individual-based model as s function of beta (not including the beta definition):

model <- function(beta)

{

pop <- data.frame(ID = c(1:n), inf.status = c(1 , rep(0, 9)) ) # Create the population

n.inf <- numeric() # Create string for number of infected

n.sus <- numeric() # Create string for number of susceptible

n.pop <- numeric() # Create string for total population

timesteps <- 1:10 # Set time steps to run the model

for(i in timesteps)

{

I <- length(pop$inf.status[pop$inf.status == 1]) # Update the infected individuals

S <- length(pop$inf.status[pop$inf.status == 0]) # Update the susceptible individuals

N <- length(pop$inf.status) # Update the total population size

PI <- 1 - exp( - beta * I / N) # Calculate the current probability of infection

new.inf <- rbinom(S , 1 , prob=PI) # Calculate which individuals will be infected

pop$inf.status[pop$inf.status == 0] <- new.inf # Assign new infections

n.inf[i] <- I # Save the infected

n.sus[i] <- S # Save the susceptible

n.pop[i] <- I + S # Save the total population

}

return(data.frame(n.pop=n.pop, n.inf=n.inf, n.sus=n.sus))

}

beta <- 0.5 # Set transmission rate

# Run the model as a function:

tmp <- model(beta)

# Make a plot of the simulations:

plot(timesteps, tmp$n.pop, type="b", ylab="Y", xlab="time",

main="Stochastic individual-based model example", ylim=c(0,10))

points(tmp$n.inf, col="red", type="b")

points(tmp$n.sus, col="blue", type="b")

# Now the model can be run several times with a new result because it is stochastic.

# We can then use the model to find the distribution of the number of infected individuals:

# We loop over a number of iterations:

iterations <- 1:1000 # Set number of iterations

infected.end <- numeric() # Create string for collecting the resulting number of infected individuals

for(j in iterations) # Loop over iterations

{

tmp <- model(beta) # Run the model and put the result in tmp

infected.end[j] <- tmp$n.inf[10] # Extract the number of infected at time step 10 (end of simulation)

}

hist(infected.end, main="Number of infected individuals at the end of the simulation")

# Tabkle of the number of simulations resulting in 1 to 10 infected individuals:

table(infected.end)

# We can then decrease the beta and simulate again:

beta <- 0.33 # transmission rate

# We loop over a number of iterations:

iterations <- 1:1000 # Set number of iterations

infected.end <- numeric() # Create string for collecting the resulting number of infected individuals

for(j in iterations) # Loop over iterations

{

tmp <- model(beta) # Run the model and put the result in tmp

infected.end[j] <- tmp$n.inf[10] # Extract the number of infected at time step 10 (end of simulation)

}

hist(infected.end, main="Number of infected individuals at the end of the simulation")

# Now the most frequent number of infected animals is around 6. Notice that there

# are a large proportion of iterations where the only infected individual is the

# one that was infected from the beginning: the epidemic never took off, or died out.

# We can see this clearly in the table:

table(infected.end)

#### Convergence ####

beta <- 0.5 # Set transmission rate

timesteps <- 1:10 # Set time steps

# We use the model defined above, in the loop.

iterations <- 1:1000 # Set number of iterations

infected.end <- numeric() # Create string for collecting the resulting number of infected individuals

for(j in iterations) # Loop over iterations

{

tmp <- model(beta) # Run the model and put the result in tmp

infected.end[j] <- tmp$n.inf[10] # Extract the number of infected at time step 10 (end of simulation)

}

# Now find the variance for 1 to 100 iterations:

conv <- numeric() # Create string for collecting the variance

for(u in 2:length(iterations)) # Loop over the number of iterations

{

conv[u] <- var(infected.end[1:u]) # Save the variance between simulation 1 to u

}

# Make a convergence plot:

plot(conv, type="l")

# Generally it seems like 400 iterations are necessary for the model to converge.
